# Supplementary material for: From sequence to enzyme mechanism using multi-label machine learning
Source: BMC Bioinformatics. 2014 May 19;15:150. doi: 10.1186/1471-2105-15-150 (PMC4229970; doi:10.1186/1471-2105-15-150)
Supplement: Additional file 2 — Java code of ml2db. Additional file ml2db_code.tar.gz contains the Java source code to run the multi-label machine learning experiments and save the results to database. The code’s Javadoc is included. [file 1471-2105-15-150-S2.zip › additional file 2/ml2db/utils/doc/index-files/index-15.html]

O-Index


JavaScript is disabled on your browser.


- Overview
- Package
- Class
- Use
- Tree
- Deprecated
- Index
- Help

- Prev Letter
- Next Letter

- Frames
- No Frames

- All Classes

A B C D E F G H I J K L M N O P Q R S T U V W X Y 


## O

objectArrayToString(Object[]) - Static method in class uk.ac.standrews.utils.main.stringutils.StringUtils


OneToMany<T,U> - Class in uk.ac.standrews.utils.main.maputils


OneToMany() - Constructor for class uk.ac.standrews.utils.main.maputils.OneToMany


OneToManyMap<T,U> - Class in uk.ac.standrews.utils.main.maputils
:   A map containing for each key a set of values.

OneToManyMap() - Constructor for class uk.ac.standrews.utils.main.maputils.OneToManyMap
:   Initialise an empty map

OneToManyMap(Vector<T>, Vector<U>) - Constructor for class uk.ac.standrews.utils.main.maputils.OneToManyMap
:   Initialise with a list of key-value pairs

oneToManyMap1() - Static method in class uk.ac.standrews.utils.test.maputils.OneToManyMapTest


oneToManyMap2() - Static method in class uk.ac.standrews.utils.test.maputils.OneToManyMapTest


oneToManyMapEmptyKeyValue() - Static method in class uk.ac.standrews.utils.test.maputils.OneToManyMapTest


OneToManyMapTest - Class in uk.ac.standrews.utils.test.maputils
:   Class

OneToManyMapTest() - Constructor for class uk.ac.standrews.utils.test.maputils.OneToManyMapTest


OptionUtils - Class in uk.ac.standrews.utils.main.guiutils
:   Utilities to manipulate command line options, main args etc.

OptionUtils() - Constructor for class uk.ac.standrews.utils.main.guiutils.OptionUtils

A B C D E F G H I J K L M N O P Q R S T U V W X Y

- Overview
- Package
- Class
- Use
- Tree
- Deprecated
- Index
- Help

- Prev Letter
- Next Letter

- Frames
- No Frames

- All Classes
